# Supplementary material for: SCAN-ACT: adoptive T cell therapy target discovery through single-cell transcriptomics
Source: Genome Med. 2025 Aug 14;17:89. doi: 10.1186/s13073-025-01514-9 (PMC12351953; doi:10.1186/s13073-025-01514-9)
Supplement: Supplementary file 2 — Additional file 2. Supplementary Figures S1-S10, portable document format file.pdf. [file 13073_2025_1514_MOESM2_ESM.pdf]

Supplementary Figures For:

SCAN-ACT: ADOPTIVE T CELL THERAPY TARGET DISCOVERY THROUGH SINGLE-CELL TRANSCRIPTOMICS

Stefano Testa[1,2], Aastha Pal[3], Ajay Subramanian[3], Sushama Varma[4], Jack Pengfei Tang[5], Danielle Graham[6], Sara Arfan[7], Minggui Pan[8], Nam Q. Bui[8], Kristen N. Ganjoo[8], Sarah Dry[9], Paul Huang[7], Matt van de Rijn[4], Wei Jiang[10], Anusha Kalbasi[3,11*], Everett J. Moding[3,11*]

[1]Department of Medicine, Stanford University, Stanford, CA, USA
[2]Current affiliation: Division of Hematology and Oncology, Beth Israel Deaconess Medical Center, Harvard Medical School, Boston, MA, USA
[3]Department of Radiation Oncology, Stanford University, Stanford, CA, USA
[4]Department of Pathology, Stanford University, Stanford, CA, USA
[5]Department of Pediatrics, Boston Children's Hospital, Boston, MA, USA
[6]Department of Surgery, Cedars-Sinai Medical Center, Los Angeles, CA, USA
[7]Division of Molecular Pathology, The Institute of Cancer Research, London, UK
[8]Division of Oncology, Department of Medicine, Stanford University, Stanford, CA, USA
[9]Department of Pathology, University of California, Los Angeles, CA, USA
[10]JWE Science Technology, Inc., Bejing, China
[11]Stanford Cancer Institute, Stanford University, Stanford, CA, USA

This document includes **Supplementary Figures S1-S10**.

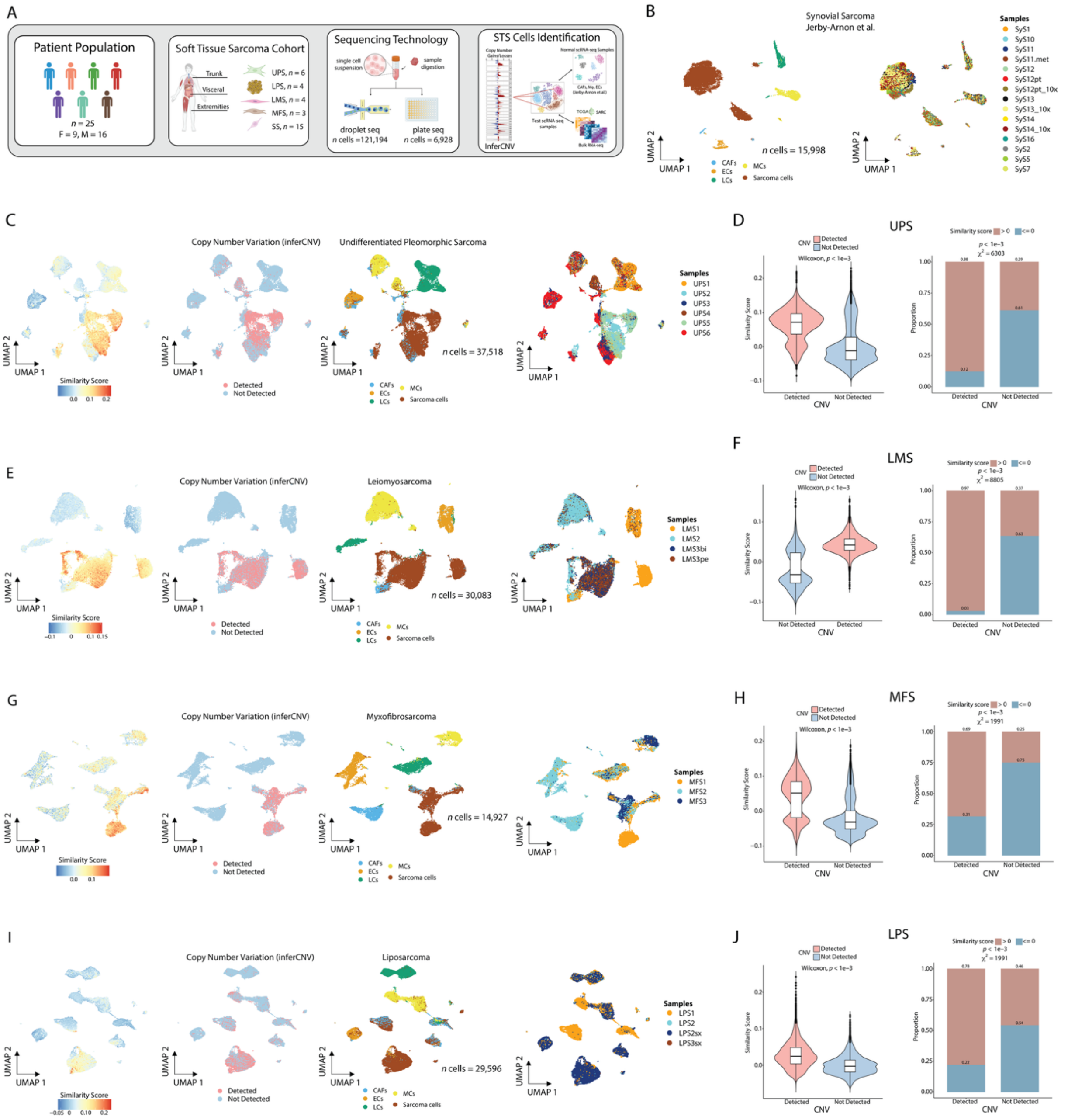
A
Patient Population
n = 25
F = 9, M = 16
Soft Tissue Sarcoma Cohort
Trunk
Visceral
Extremities
UPS, n = 6
LPS, n = 4
LMS, n = 4
MFS, n = 3
SS, n = 15
Sequencing Technology
single cell suspension
sample digestion
droplet seq
n cells = 121,194
plate seq
n cells = 6,928
STS Cells Identification
InferCNV
B
Synovial Sarcoma
Jerby-Arnon et al.
n cells = 15,998
CAFs
ECs
LCs
MCs
Sarcoma cells
Samples
SyS1
SyS10
SyS11
SyS11.met
SyS12
SyS12pt
SyS12pt_10x
SyS13
SyS13_10x
SyS14
SyS14_10x
SyS16
SyS2
SyS5
SyS7
C
Copy Number Variation (inferCNV)
Undifferentiated Pleomorphic Sarcoma
n cells = 37,518
Similarity Score
Detected
Not Detected
Samples
UPS1
UPS2
UPS3
UPS4
UPS5
UPS6
D
UPS
Wilcoxon, p < 1e−3
Similarity score > 0 <= 0
p < 1e−3
χ² = 6303
E
Copy Number Variation (inferCNV)
Leiomyosarcoma
n cells = 30,083
Samples
LMS1
LMS2
LMS3bi
LMS3pe
F
LMS
χ² = 8805
G
Copy Number Variation (inferCNV)
Myxofibrosarcoma
n cells = 14,927
Samples
MFS1
MFS2
MFS3
H
MFS
χ² = 1991
I
Copy Number Variation (inferCNV)
Liposarcoma
n cells = 29,596
Samples
LPS1
LPS2
LPS2sx
LPS3sx
J
LPS
χ² = 1991
UMAP 1
UMAP 2
CNV
Similarity Score
Proportion

Fig S1. **Single cell RNA sequencing of soft tissue sarcoma patient samples**.

(A) Scheme illustrating scRNA-seq of STS sarcoma samples and tumor cell annotation strategy. **(C, E, G, I)**. UMAP plots showing the samples in the study with cells colored based on the transcriptomic similarity score, CNV status, cell identity, or by sample. **(B)** UMAP plot showing cell identity annotation for the SS samples. **(D**, **F**, **H**, **J)** Violin plots showing the distribution of the similarity score (left) and stacked bar charts showing the proportion of cells with similarity score > 0 and ≤ 0 (right) for CD45- cells with and without CNVs detected by inferCNV in the UPS (**D**), LMS (**F**), MFS (**J**), and LPS (**H**) samples. A Wilcoxon rank-sum test was used to compare the two distributions in the violin plots and a Chi-square test was used to compare proportions in the bar charts.

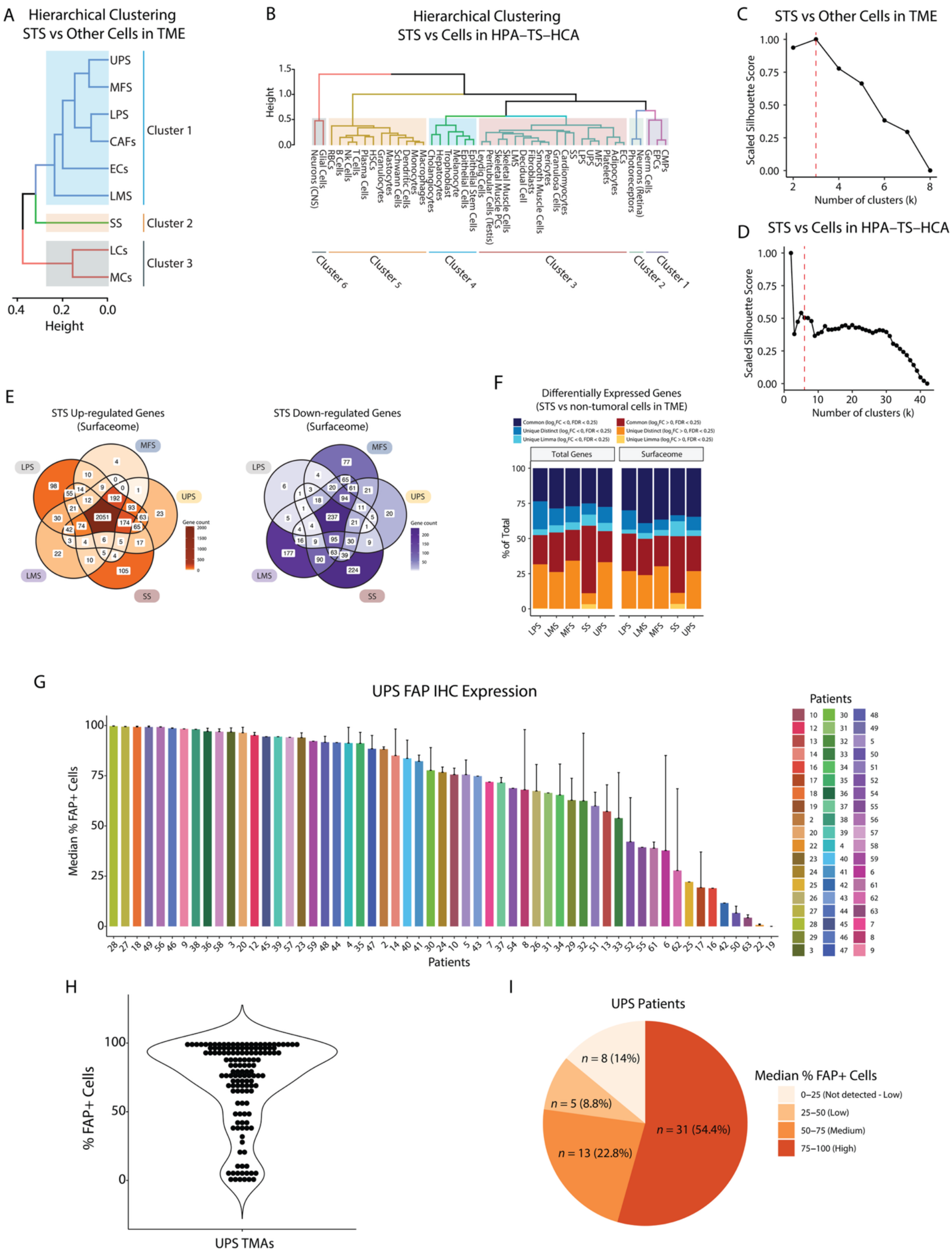
A
Hierarchical Clustering
STS vs Other Cells in TME
UPS
MFS
LPS
CAFs
ECs
LMS
SS
LCs
MCs
Cluster 1
Cluster 2
Cluster 3
0.4
0.2
0.0
Height
B
Hierarchical Clustering
STS vs Cells in HPA–TS–HCA
Height
1.5
1.0
0.5
0.0
Neurons (CNS)
Glial Cells
RBCs
B Cells
NK Cells
T Cells
Plasma Cells
HSCs
Granulocytes
Mastocytes
Schwann Cells
Dendritic Cells
Macrophages
Monocytes
Cholangiocytes
Hepatocytes
Trophoblast
Melanocyte
Epithelial Cells
Epithelial Stem Cells
Leydig Cells
Peritubular Cells (Testis)
LMS
Skeletal Muscle PCs
Skeletal Muscle Cells
Fibroblasts
Decidual Cell
Pericytes
Smooth Muscle Cells
Granulosa Cells
Cardiomyocytes
SS
LPS
UPS
MFS
Platelets
Adipocytes
ECs
Photoreceptors
Neurons (Retina)
Germ Cells
EPCs
CMPs
Cluster 6
Cluster 5
Cluster 4
Cluster 3
Cluster 2
Cluster 1
C
STS vs Other Cells in TME
Scaled Silhouette Score
1.00
0.75
0.50
0.25
0.00
2
4
6
8
Number of clusters (k)
D
STS vs Cells in HPA–TS–HCA
Scaled Silhouette Score
1.00
0.75
0.50
0.25
0.00
0
10
20
30
40
Number of clusters (k)
E
STS Up-regulated Genes
(Surfaceome)
LPS
MFS
UPS
LMS
SS
Gene count
STS Down-regulated Genes
(Surfaceome)
F
Differentially Expressed Genes
(STS vs non-tumoral cells in TME)
Total Genes
Surfaceome
% of Total
100
75
50
25
0
LPS
LMS
MFS
SS
UPS
G
UPS FAP IHC Expression
Median % FAP+ Cells
100
75
50
25
0
Patients
H
% FAP+ Cells
100
50
0
UPS TMAs
I
UPS Patients
n = 8 (14%)
n = 5 (8.8%)
n = 13 (22.8%)
n = 31 (54.4%)
Median % FAP+ Cells
0–25 (Not detected - Low)
25–50 (Low)
50–75 (Medium)
75–100 (High)

Fig S2. Analysis of differentially expressed genes in STS and FAP IHC expression in UPS.

(A) Results of unsupervised hierarchical clustering of STS versus non-tumoral cells in the TME. The clustering analysis identified three cell clusters. **(B)** Results of unsupervised hierarchical clustering of STS versus normal cells in HPA-TS-HCA. The clustering analysis identified six cell clusters. **(C)** Plot showing the variation of the silhouette score for different number of clusters (k) for the comparisons of STS versus non-tumoral cells in the TME. **(D)** Plot showing the variation of the silhouette score for different number of clusters (k) for the comparisons of STS versus cells in the HPA-TS-HCA. **(E)** Venn diagrams showing the number of STS up-regulated and down-regulated genes shared between histotypes considering only surfaceome genes. **(F)** Stacked bar chart showing the fraction of DEGs in STS versus non-tumoral cells in the TME, identified with Limma-voom, distinct, or both methods, considering surfaceome and total genes separately. **(G)** Bar chart showing the median % of cells expressing FAP as measured through IHC on 138 TMAs from 57 UPS patients. **(H)** Violin plot showing the distribution of the % of FAP^+ cells in the 138 UPS TMAs obtained from 57 patients. **(I)** Pie chart showing the fraction of patients with Not Detected-Low FAP expression (0-25% FAP^+ cells), Low expression (25-50 % FAP^+ cells), Medium expression (50-75 % FAP^+ cells), and High expression (75-100 % FAP^+ cells). Expression is measured as the median value considering all the TMAs for each patient.

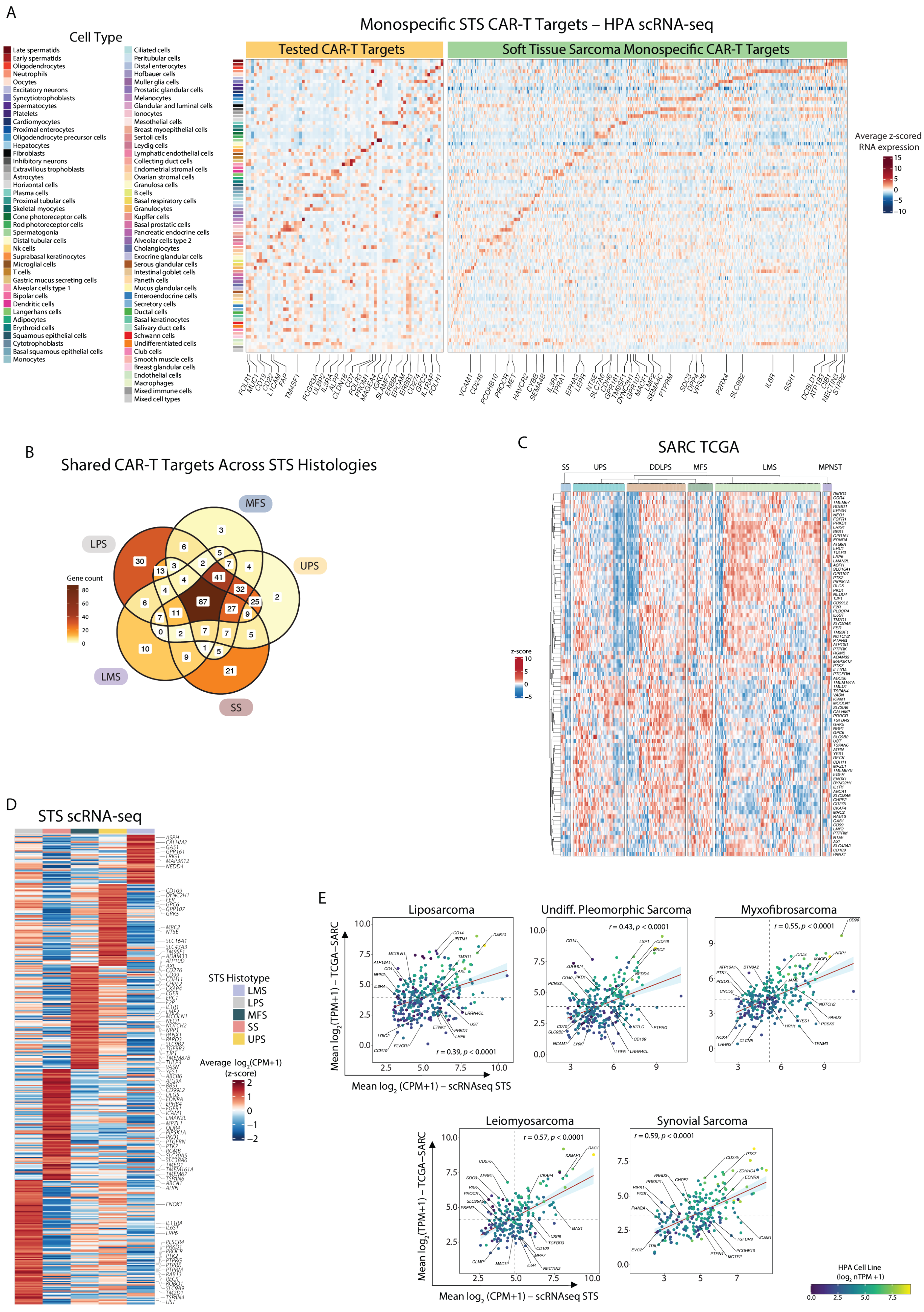

A
Monospecific STS CAR-T Targets – HPA scRNA-seq
Cell Type
Tested CAR-T Targets
Soft Tissue Sarcoma Monospecific CAR-T Targets
Average z-scored RNA expression
B
Shared CAR-T Targets Across STS Histologies
MFS
LPS
UPS
LMS
SS
Gene count
C
SARC TCGA
SS
UPS
DDLPS
MFS
LMS
MPNST
z-score
D
STS scRNA-seq
STS Histotype
LMS
LPS
MFS
SS
UPS
Average log2(CPM+1) (z-score)
E
Liposarcoma
Undiff. Pleomorphic Sarcoma
Myxofibrosarcoma
Leiomyosarcoma
Synovial Sarcoma
r = 0.39, p < 0.0001
r = 0.43, p < 0.0001
r = 0.55, p < 0.0001
r = 0.57, p < 0.0001
r = 0.59, p < 0.0001
Mean log2(TPM+1) – TCGA–SARC
Mean log2 (CPM+1) – scRNAseq STS
HPA Cell Line (log2 nTPM +1)

Fig S3. **Monospecific CAR-T targets**.

(A) scRNA-seq expression of monospecific CAR targets in the HPA scRNA-seq database. Heatmap shows the average z-scored and median-centered pseudo-bulk RNA expression for tested CAR-T cells targets (upper panel) and the monospecific STS CAR targets identified in this study (bottom panel) as measured in the HPA scRNA-seq database. RNA expression values were first pseudo-bulked by cell cluster, and then averaged across clusters and tissues for a given cell type. **(B)** Venn diagram showing the number of targets shared between the five STS histotypes. **(C)** Expression of the monospecific CAR targets common to MFS, LMS, SS, LPS, and UPS in TCGA SARC. RNA expression in $\log_2$(TPM+1) was median-centered across samples and z-scored across genes before plotting. **(D)** Expression of the candidate monospecific CAR targets in scRNA-seq STS samples. RNA expression in $\log_2$(CPM+1) was averaged across cells of the same type before median-centering and z-scoring. **(E)** Pearson correlation between average scRNA-seq expression and average bulk RNA-seq in matched STS samples from TCGA SARC, for CAR-T targets in each STS. Dots are colored based on gene expression in HPA sarcoma cell lines. CMPs: Common Myeloid Progenitor Cells; EPCs: Erythroid Progenitor Cells; HSCs: Hematopoietic Stem Cells; RBCs: Red blood cells; ECs: Endothelial Cells; MCs: Myeloid Cells; LCs: Lymphoid Cells; CAFs: Cancer associated fibroblasts.

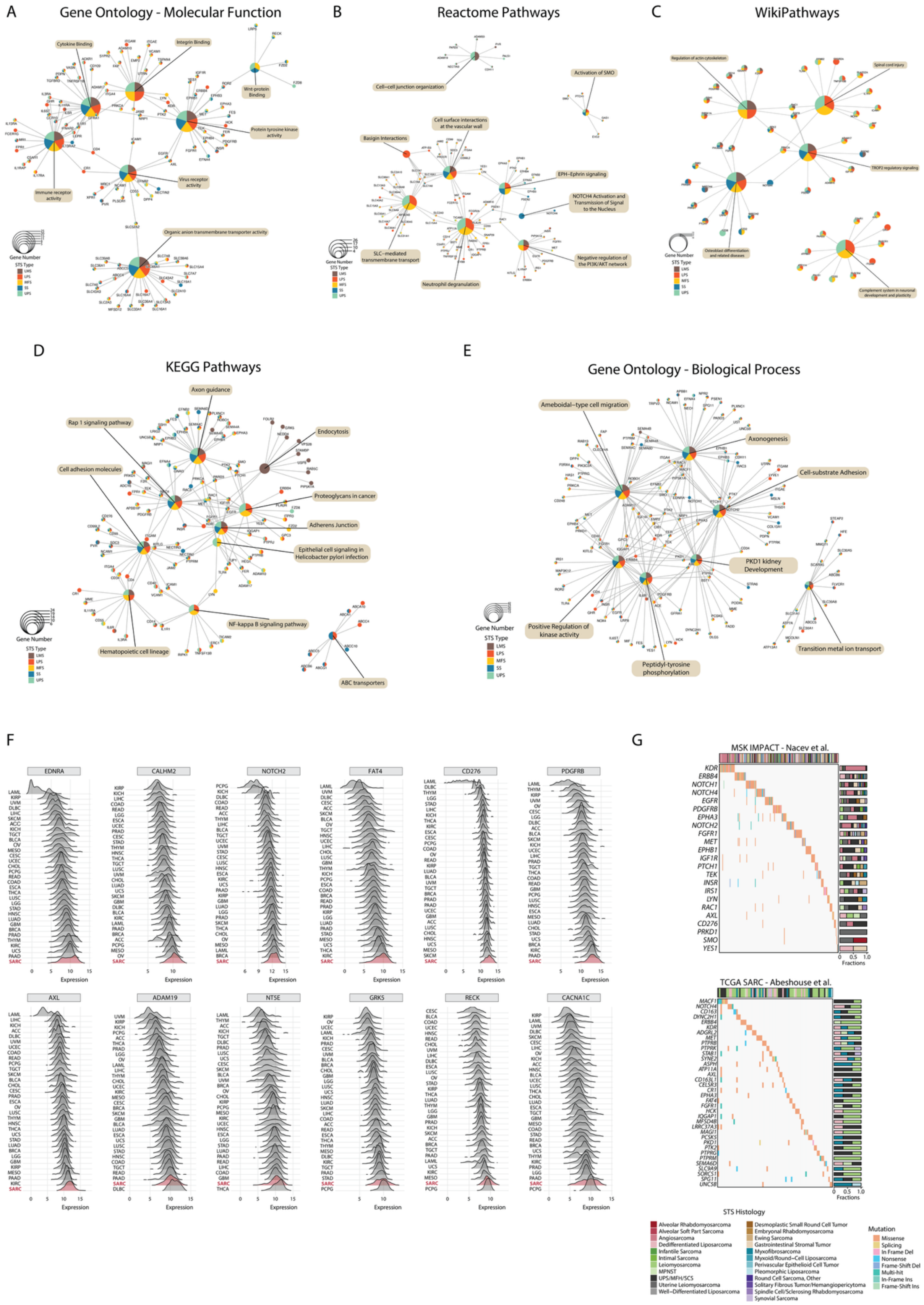

A
Gene Ontology - Molecular Function
Cytokine Binding
Integrin Binding
Wnt-protein Binding
Protein tyrosine kinase activity
Immune receptor activity
Virus receptor activity
Organic anion transmembrane transporter activity
B
Reactome Pathways
Cell−cell junction organization
Activation of SMO
Cell surface interactions at the vascular wall
Basigin Interactions
EPH−Ephrin signaling
NOTCH4 Activation and Transmission of Signal to the Nucleus
SLC−mediated transmembrane transport
Negative regulation of the PI3K/AKT network
Neutrophil degranulation
C
WikiPathways
Regulation of actin cytoskeleton
Spinal cord injury
TROP2 regulatory signaling
Osteoblast differentiation and related diseases
Complement system in neuronal development and plasticity
D
KEGG Pathways
Axon guidance
Rap 1 signaling pathway
Endocytosis
Cell adhesion molecules
Proteoglycans in cancer
Adherens Junction
Epithelial cell signaling in Helicobacter pylori infection
NF-kappa B signaling pathway
Hematopoietic cell lineage
ABC transporters
E
Gene Ontology - Biological Process
Ameboidal−type cell migration
Axonogenesis
Cell-substrate Adhesion
PKD1 kidney Development
Positive Regulation of kinase activity
Peptidyl-tyrosine phosphorylation
Transition metal ion transport
Gene Number
STS Type
LMS
LPS
MFS
SS
UPS
F
EDNRA
CALHM2
NOTCH2
FAT4
CD276
PDGFRB
AXL
ADAM19
NT5E
GRK5
RECK
CACNA1C
SARC
Expression
G
MSK IMPACT - Nacev et al.
TCGA SARC - Abeshouse et al.
Fractions
STS Histology
Alveolar Rhabdomyosarcoma
Alveolar Soft Part Sarcoma
Angiosarcoma
Dedifferentiated Liposarcoma
Infantile Sarcoma
Intimal Sarcoma
Leiomyosarcoma
MPNST
UPS/MFH/SCS
Uterine Leiomyosarcoma
Well−Differentiated Liposarcoma
Desmoplastic Small Round Cell Tumor
Embryonal Rhabdomyosarcoma
Ewing Sarcoma
Gastrointestinal Stromal Tumor
Myxofibrosarcoma
Myxoid/Round−Cell Liposarcoma
Perivascular Epithelioid Cell Tumor
Pleomorphic Liposarcoma
Round Cell Sarcoma, Other
Solitary Fibrous Tumor/Hemangiopericytoma
Spindle Cell/Sclerosing Rhabdomyosarcoma
Synovial Sarcoma
Mutation
Missense
Splicing
In Frame Del
Nonsense
Frame-Shift Del
Multi-hit
In-Frame Ins
Frame-Shift Ins

Fig S4. **Gene set over-representation analysis (ORA) of monospecific CAR-T targets by sarcoma histotype, TCGA Pan-Cancer Atlas expression for selected monospecific CAR-T targets, and incidence of SNV/Indels for monospecific CAR-T targets in TCGA and MSK-IMPACT.**

(A-E) Gene concept networks showing results of gene set ORA using **(A)** GO Molecular Function terms **(B)**, Kyoto Encyclopedia of Genes and Genomes (KEGG) pathways, **(C)** WikiPathways terms, **(D)** Reactome pathways, and GO Biological Process terms **(E)**. **(F)** Ridge plots showing RNA expression in $\log_2$(TPM+1) across different cancers in the Pan-Cancer Atlas from TCGA for selected monospecific CAR-T cells targets. Soft tissue sarcoma samples (SARC) are highlighted in bold red. **(G)** Oncoplots showing recurring SNVs/Indels for monospecific CAR-T targets in MSK-IMPACT (left) and TCGA SARC (right).

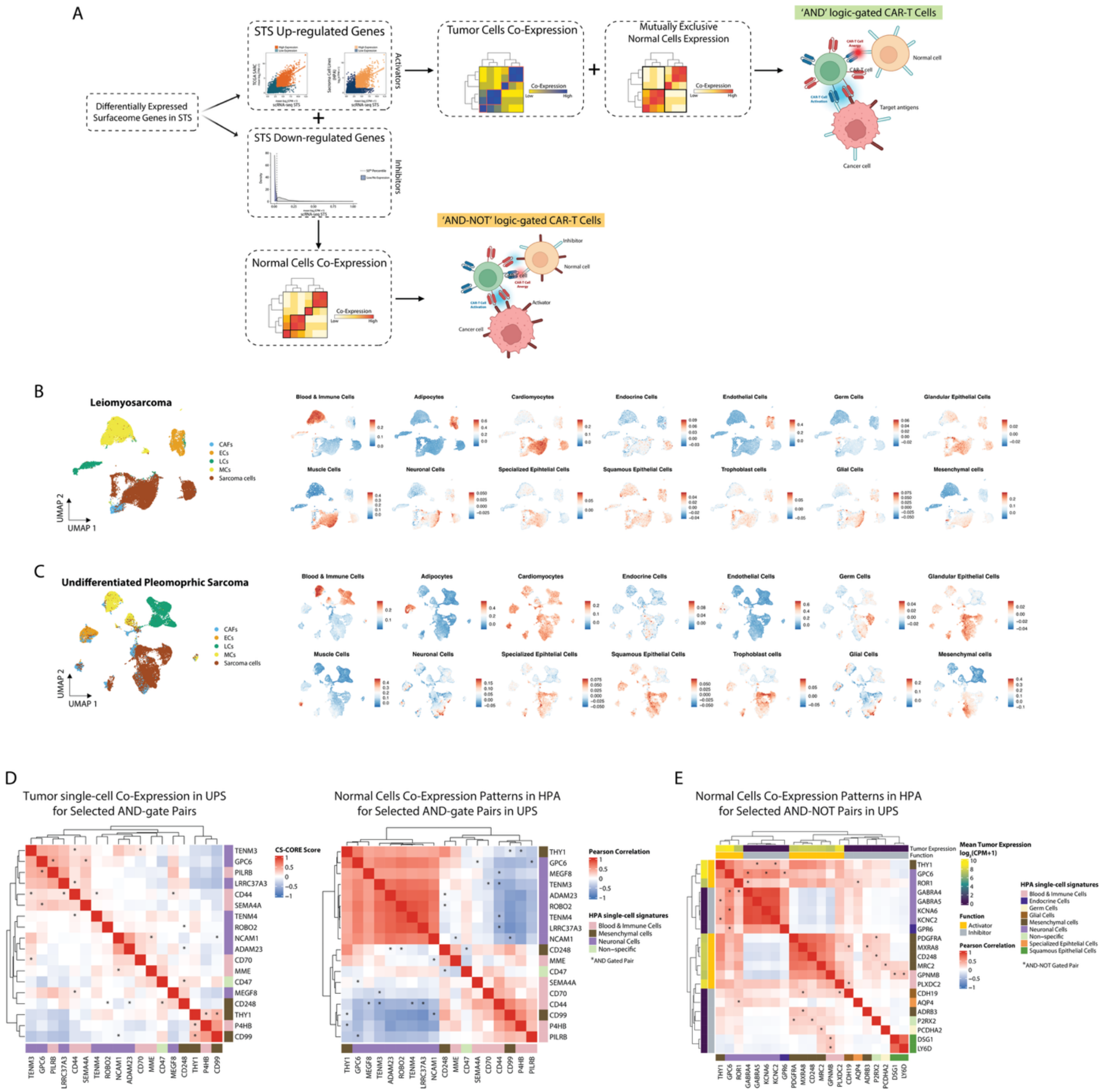

Fig S5. **Bispecific CAR-T cell targets in STS**.

(A) Illustration showing the steps of the bispecific CAR targets pair discovery pipeline. **(B, C)** UMAP plots showing the expression of cell-specific gene signatures in LMS **(B)**, and UPS **(C)**. The gene signatures used represent all the genes belonging to a given HPA scRNA-seq cell type-specific signature. **(D)** Heatmaps showing the single-cell tumor co-expression as computed through CS-CORE for selected 'AND'-gate target pairs in UPS (*left*), and the

Pearson correlation across cell types in the HPA single cell RNA-seq dataset for selected 'AND' CAR-T pairs in UPS (*right*). Rows and columns are annotated based on the HPA single-cell signature to which each gene belongs to. Notable pairs are indicated with an asterisk. (**E**) Heatmap showing Pearson correlation across normal cell types in HPA single cell RNA-seq dataset for selected 'AND-NOT' pairs in UPS. Rows and columns are annotated based on the HPA single-cell signature to which each gene belongs to. Notable pairs are indicated with an asterisk.

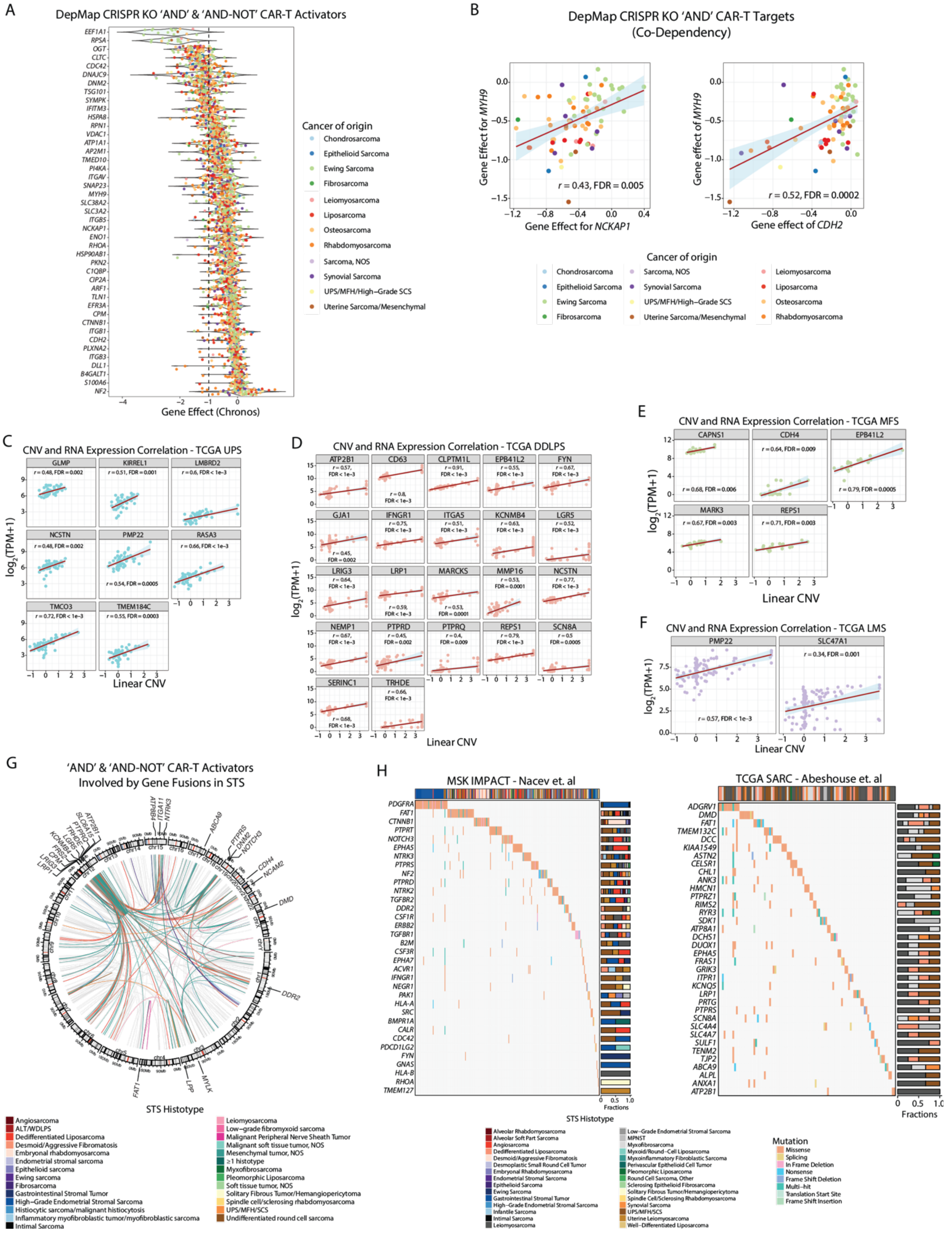
A
DepMap CRISPR KO 'AND' & 'AND-NOT' CAR-T Activators
Gene Effect (Chronos)
Cancer of origin
Chondrosarcoma
Epithelioid Sarcoma
Ewing Sarcoma
Fibrosarcoma
Leiomyosarcoma
Liposarcoma
Osteosarcoma
Rhabdomyosarcoma
Sarcoma, NOS
Synovial Sarcoma
UPS/MFH/High-Grade SCS
Uterine Sarcoma/Mesenchymal
B
DepMap CRISPR KO 'AND' CAR-T Targets
(Co-Dependency)
Gene Effect for MYH9
Gene Effect for NCKAP1
r = 0.43, FDR = 0.005
Gene effect of MYH9
Gene effect of CDH2
r = 0.52, FDR = 0.0002
C
CNV and RNA Expression Correlation - TCGA UPS
D
CNV and RNA Expression Correlation - TCGA DDLPS
E
CNV and RNA Expression Correlation - TCGA MFS
F
CNV and RNA Expression Correlation - TCGA LMS
log2(TPM+1)
Linear CNV
G
'AND' & 'AND-NOT' CAR-T Activators
Involved by Gene Fusions in STS
STS Histotype
H
MSK IMPACT - Nacev et. al
TCGA SARC - Abeshouse et. al
Fractions
STS Histotype
Mutation
Missense
Splicing
In Frame Deletion
Nonsense
Frame Shift Deletion
Multi-hit
Translation Start Site
Frame Shift Insertion

Fig S6. Functional characterization of bispecific CAR-T cell targets in STS.

(A) Violin plot showing the effect on cell growth of the knock-out (KO) of 'AND' CAR targets and 'AND-NOT' CAR activators in sarcoma cell lines as reported in the DepMap public database. Only targets that are essential for cell growth in at least one sarcoma cell line are shown. The gene KO effect is quantified based on the Chronos algorithm with more negative values corresponding to genes that are more essential for cell growth. The vertical dashed line intersecting at –1 indicates the median Chronos value for the known essential genes. **(B)** Scatter plots showing CRISPR KO co-dependency plots for the 'AND' CAR pairs *MYH9-NCKAP* (left) and *MYH9-NCKAP* (right). Pearson correlation was used, and the p-values were corrected for multiple hypothesis testing using the BH method with significance set to FDR $<$ 0.01. **(C**, **D**, **E**, **F)** Scatter plots showing the Pearson correlation between linear CNV level and RNA expression for UPS (**C**), DDLPS (**D**), MFS (**E**), LMS (**F**) samples in TCGA for selected 'AND' CAR and 'AND-NOT' CAR (activators) targets. RNA expression is reported in $\log_2$(TPM+1). The p-values shown were adjusted for multiple hypothesis testing using the BH method with significance set to FDR $<$ 0.01. **(G)** Circos plot showing gene fusions in STS from the Mitelman database, MSK-IMPACT, and TCGA SARC cohorts, for which one of the involved genes is a bispecific CAR target. Colored links show fusions involving the top 30 genes most recurrently involved in gene fusions among all bispecific CAR targets. The link colors indicate the STS histotype in which the fusion has been identified. **(H)** Oncoplots showing recurring SNVs/Indels for bispecific CAR targets in the MSK-IMPACT (*left*) and TCGA cohorts (*right*). The stacked bar charts show the mutation frequency per each gene across different soft tissue sarcoma histologies. Only the most frequently mutated genes across all samples in each cohort are shown. ALT: Atypical Lyopmatous Tumor; WDLPS: Well-differentiated Liposarcoma; GIST: Gastrointestinal Stromal Tumor; MPNST: Malignant Peripheral Nerve Sheath Tumor; IMT: Inflammatory Myofibroblastic Tumor; UPS:

Undifferentiated Pleomorphic Sarcoma; MFH: Malignant Fibrous Histiocytoma; SCS: High-grade Spindle Cell Sarcoma.

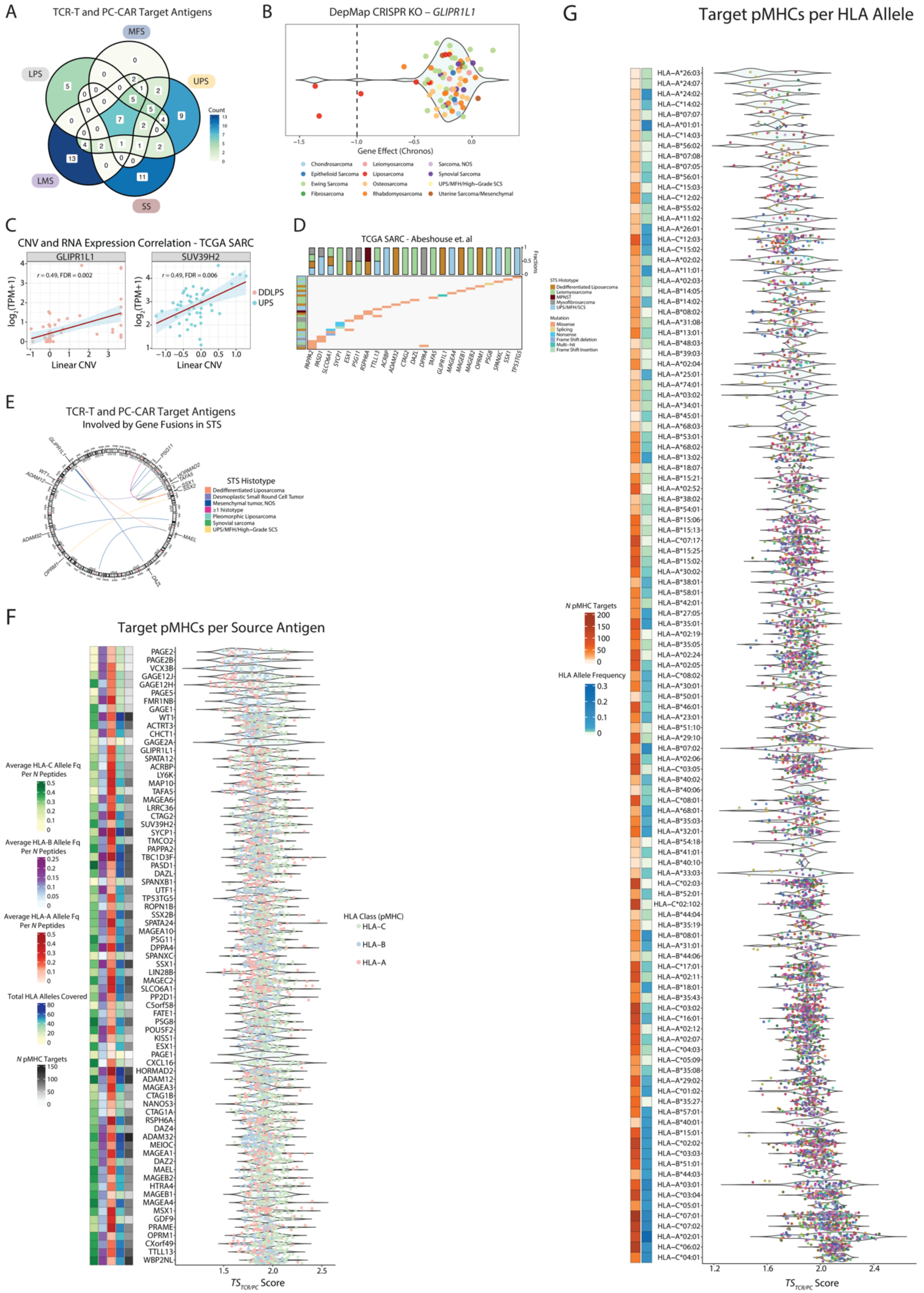
A
TCR-T and PC-CAR Target Antigens
MFS
LPS
UPS
LMS
SS
Count
B
DepMap CRISPR KO – GLIPR1L1
Gene Effect (Chronos)
Chondrosarcoma
Epithelioid Sarcoma
Ewing Sarcoma
Fibrosarcoma
Leiomyosarcoma
Liposarcoma
Osteosarcoma
Rhabdomyosarcoma
Sarcoma, NOS
Synovial Sarcoma
UPS/MFH/High-Grade SCS
Uterine Sarcoma/Mesenchymal
C
CNV and RNA Expression Correlation - TCGA SARC
GLIPR1L1
SUV39H2
r = 0.49, FDR = 0.002
r = 0.49, FDR = 0.006
DDLPS
UPS
log2(TPM+1)
Linear CNV
D
TCGA SARC - Abeshouse et. al
Fractions
STS Histotype
Dedifferentiated Liposarcoma
Leiomyosarcoma
MPNST
Myxofibrosarcoma
UPS/MFH/SCS
Mutation
Missense
Splicing
Nonsense
Frame Shift deletion
Multi_hit
Frame Shift Insertion
E
TCR-T and PC-CAR Target Antigens
Involved by Gene Fusions in STS
STS Histotype
Dedifferentiated Liposarcoma
Desmoplastic Small Round Cell Tumor
Mesenchymal tumor, NOS
≥1 histotype
Pleomorphic Liposarcoma
Synovial sarcoma
UPS/MFH/High-Grade SCS
GLIPR1L1
PSG11
HORMAD2
WT1
ADAM12
ADAM32
MAEL
OPRM1
DAZL
F
Target pMHCs per Source Antigen
Average HLA-C Allele Fq Per N Peptides
Average HLA-B Allele Fq Per N Peptides
Average HLA-A Allele Fq Per N Peptides
Total HLA Alleles Covered
N pMHC Targets
PAGE2
PAGE2B
VCX3B
GAGE12J
GAGE12H
PAGE5
FMR1NB
GAGE1
WT1
ACTRT3
CHCT1
GAGE2A
GLIPR1L1
SPATA12
ACRBP
LY6K
MAP10
TAFA5
MAGEA6
LRRC36
CTAG2
SUV39H2
SYCP1
TMCO2
PAPPA2
TBC1D3F
PASD1
DAZL
SPANXB1
UTF1
TP53TG5
ROPN1B
SSX2B
SPATA24
MAGEA10
PSG11
DPPA4
SPANXC
SSX1
LIN28B
MAGEC2
SLCO6A1
PP2D1
C5orf58
FATE1
PSG8
POU5F2
KISS1
ESX1
PAGE1
CXCL16
HORMAD2
ADAM12
MAGEA3
CTAG1B
NANOS3
CTAG1A
RSPH6A
DAZ4
ADAM32
MEIOC
MAGEA1
DAZ2
MAEL
MAGEB2
HTRA4
MAGEB1
MAGEA4
MSX1
GDF9
PRAME
OPRM1
CXorf49
TTLL13
WBP2NL
HLA Class (pMHC)
HLA-C
HLA-B
HLA-A
TS TCR/PC Score
G
Target pMHCs per HLA Allele
N pMHC Targets
HLA Allele Frequency
Source Antigen (pMHC)
TS TCR/PC Score

Fig S7. **Characterization of TCR-T and PC-CAR target antigens and derived target pMHCs**.

(A) Venn diagram showing the number of PC-CAR and TCR-T target antigens shared between the different STS studied. **(B)** Violin plot showing the CRISPR KO effect of *GLIPR1L1* on STS cell lines. The gene KO effect is quantified using the Chronos algorithm with more negative values corresponding to genes more essential for cell growth. The vertical dashed line intersecting at –1 indicates the median Chronos value for known essential genes. Dots are colored based on the cell line cancer of origin. **(C)** Scatter plots showing Pearson correlation between CNA level and RNA expression in TCGA DDLPS and UPS samples for *GLIPR1L1* and *SUV39H2*, respectively. P-values were adjusted for multiple hypothesis testing using the BH method, and significance was set at FDR < 0.01. **(D)** Oncoplot showing recurring SNVs/Indels for TCR-T and PC-CAR target antigens in TCGA SARC. The stacked bar charts show the mutation frequency per each gene across different STS histotypes. Only the most frequently mutated genes across all samples are shown. **(E)** Circos plot showing gene fusions in STS from the Mitelman database, MSK-IMPACT and TCGA SARC, for which one of the involved genes is a TCR-T and PC-CAR target antigen. The link colors indicate the STS histotype in which the fusion was identified. **(F)** Violin plot showing the distribution of $TS_{TCR/PC}$ scores for the candidate $pMHCs$ selected for each antigen. Dots are colored based on the HLA class restriction of each $pMHC$. The heatmap on the left shows the total number of target $pMHCs$ per antigen, the total number of HLA alleles covered, and the cumulative HLA-A, HLA-B, and HLA-C allele frequency for each antigen. The cumulative allele frequencies for each HLA locus were calculated by first isolating all $pMHC$ pairs that bind to HLA alleles of a given locus (A, B, or C) for each antigen. Then, for each locus, the HLA allele frequencies of these $pMHCs$ were summed and divided by the number of unique peptides that form all the $pMHCs$ binding to HLA alleles of that locus. **(G)** Violin plot showing the distribution

of $TS_{TCR/PC}$ scores for the candidate $pMHCs$ grouped by their HLA allele restriction. The dots are colored based on the source tumor antigen of the $pMHCs$. The heatmap on the left shows the total number of $pMHCs$ per each HLA allele, and the average worldwide allele frequency for each HLA allele.

A

Peptide Similarity Analysis of Known Cross-Reactive Peptide Pairs

Source Antigen	Target Peptide	Cross-Reactive Peptide	Normalized PMBEC Similarity	Normalized AAindex Similarity	Peptide Similarity Score	Cross-Reactive Peptide Organism	HLA
MAGEA-6	YIFATCLGLS	YIFAACLLLI	1	1	2	Mycobactermium Penetrantis	HLA-A*02:01
C1QTNF12	LLGPQLVLL	LLGPLLVLL	0.908869595	0.88228284	1.791152436	HBV	HLA-A*02:01
EPCAM	VVAGIVVLV	VVAGIIALV	0.913012115	0.770224445	1.68323656	HIV-1	HLA-A*02:01
MDK	ALLALTSAV	ALMAFTSAV	0.912451862	0.766779325	1.679231188	HCV	HLA-A*02:01
MDK	LLLTLLALL	LLLTLLLLL	0.789303717	0.884015257	1.673318974	Adenovirus	HLA-A*02:01
DPYSL2	IPRRTTQRI	IPRRIRQRI	0.8906457	0.769828303	1.660474003	HIV-1	HLA-B*07:02
gp100	MLGTHTMEV	MLGTHAMLV	0.8710389	0.770499778	1.641538678	HCMV	HLA-A*02:01
NDUFS2	VSDGSSRPY	SSDNSSRPY	0.869510415	0.767433998	1.636944413	HIV-1	HLA-A*01:01
HEPACAM	RLAPFVYLL	RLAPFGYKI	0.851321611	0.677718908	1.529040519	Encephalitis virus	HLA-A*02:01
CD274	LLNAFTVTV	LLNAFAIAV	0.86741129	0.65556493	1.52297622	HIV-1	HLA-A*02:01
TERT	RLVDDFLLV	RLVDEFLAI	0.836655457	0.679907523	1.516562981	HIV-1	HLA-A*02:01
ISG15	MLAGNEFQV	MLAGNAFTA	0.81887112	0.680004889	1.498876008	Calicivirus	HLA-A*02:01
MART-1	AAGIGILTV	IAGIGILAI	0.772540075	0.679898621	1.452438696	Pseudorabies	HLA-A*02:01
CEA	IMIGVLVGV	IMVGALIGV	0.791737024	0.650953595	1.442690619	HIV-1	HLA-A*02:01
MART-1	AAGIGILTV	GAGIGVLTA	0.731202806	0.682462736	1.413665542	Bacillus Polymyxa	HLA-A*02:01
MART-1	AAGIGILTV	QAGIGILLA	0.720063659	0.679487922	1.399551581	E. Coli	HLA-A*02:01
CNIH4	LLNLPVATW	HCNLSVATW	0.665910794	0.674457652	1.340368446	HIV-1	HLA-B*58:01
CCR9	AIADLLFLV	RLRDLLFLV	0.613536037	0.677826374	1.291362411	HIV-1	HLA-A*02:01
TMEM161A	ALGGLLTPL	CLGGLLTMV	0.551620235	0.686133548	1.237753783	EBV	HLA-A*02:01
TMEM161A	ALGGLLTPL	LLGGLLTMV	0.530080979	0.686820687	1.216901666	E. Coli	HLA-A*02:01
MAGEA-3	EVDPIGHLY	ESDPIVAQY	0.649505777	0.54274076	1.192246536	Homo Sapiens (Titin)	HLA-A*01:01
MART-1	AAGIGILTV	GAGIGVAVL	0.59185572	0.457640834	1.049496554	HSV-2	HLA-A*02:01
MART-1	AAGIGILTV	GIGIGVLAA	0.559592626	0.472963967	1.032556593	HSV-1	HLA-A*02:01
MART-1	AAGIGILTV	LIVIGILIL	0.521802407	0.475771354	0.997573761	Adenovirus	HLA-A*02:01
MART-1	EAAGIGILTV	MLSGIGIFFI	0.523095177	0.425643599	0.948738776	Chlamydia Trachomatis	HLA-A*02:01

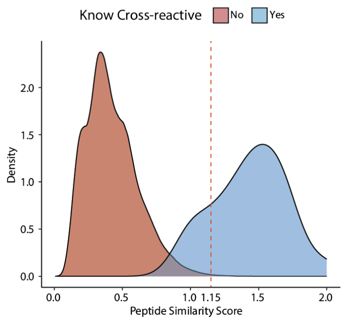

B

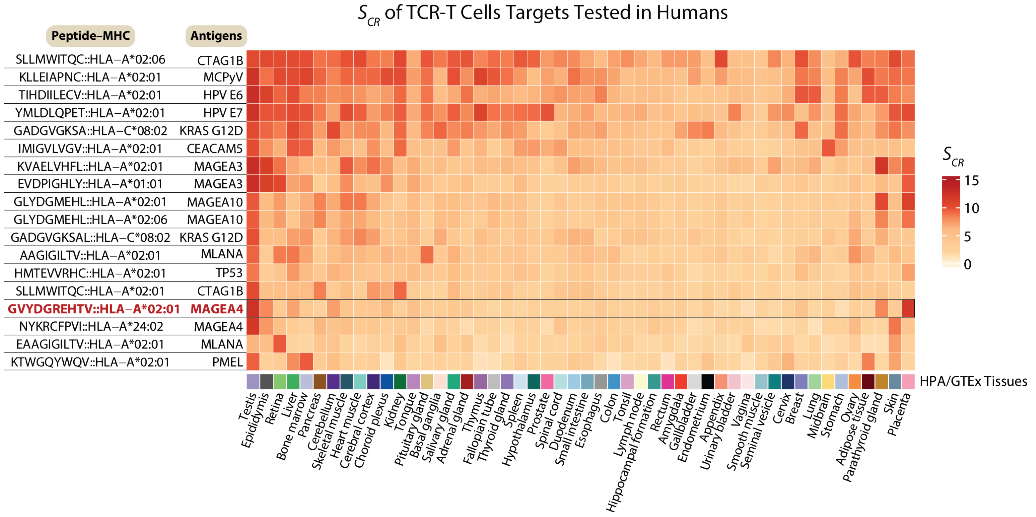

C

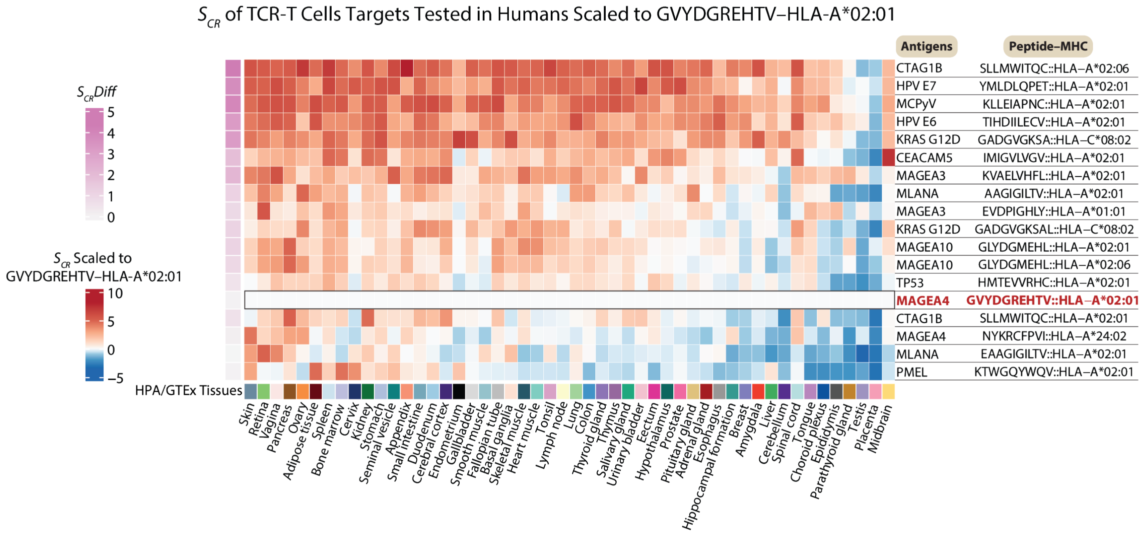

Fig S8. **Validation of peptide similarity and normal tissue cross-reactivity algorithms**. **(A)** The table on the *left* shows the results of the peptide similarity analysis on known pairs of cross-reactive peptides. The density plot on the *right* shows the distribution of the peptide similarity scores (S_{sim}) obtained between known cross-reactive peptide pairs (blue curve) and those obtained between each tested peptide and all the peptides of same length binding to the same HLA allele as reported in the normal ligandome (red curve). The vertical line shows the S_{sim} score cutoff of 1.15, used to define highly similar peptides as it provided the highest specificity in identifying known cross-reactive peptides. **(B)** Heatmap showing the HPA/GTEx tissue-specific S_{CR} scores for $pMHCs$ targeted by TCR-T cells that have been tested in clinical trials, and for which safety outcomes are available. For each $pMHC$, the parent antigen is displayed. Afami-cel's target, GVYDGREHTV–HLA-A*02:01, is highlighted for comparison. **(C)** Heatmap showing the HPA/GTEx tissue-specific S_{CR} scores for each known $pMHC$ target scaled to the S_{CR} scores of afami-cel's target, GVYDGREHTV–HLA-A*02:01, and obtained as follows: $\left(\frac{\left(S_{CR}^{j}(target)-S_{CR}^{j}(reference)\right)}{\sigma_j}\right) \times 2\ if\ S_{CR}^{j}(target) > S_{CR}^{j}(reference)$, where σ_j is the standard deviation of the S_{CR} values for all the target $pMHCs$ in the jth tissue. $S_{CR}Diff$: differential S_{CR} profile compared to afami-cel's target, GVYDGREHTV–HLA-A*02:01.

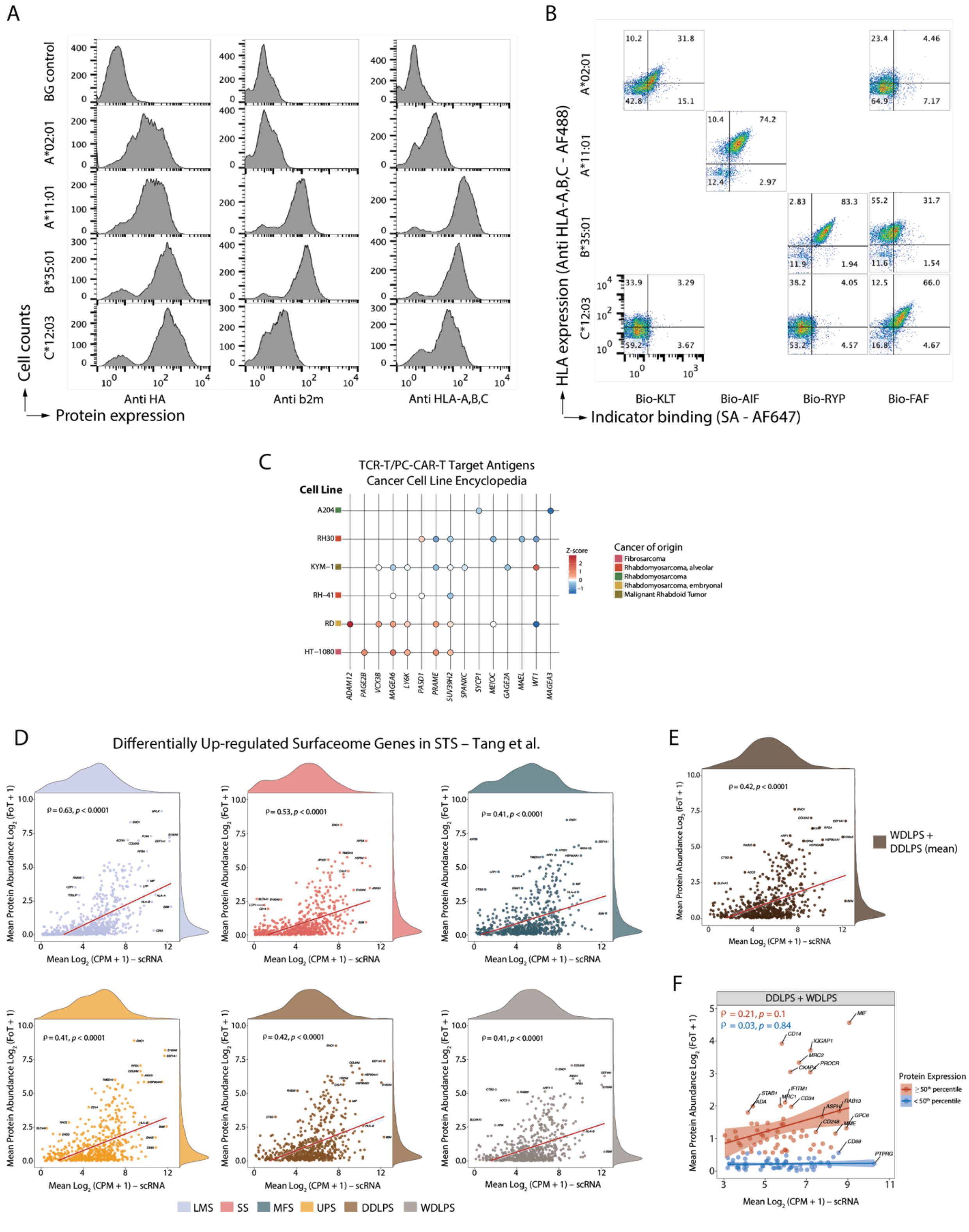
A
B
C
D
E
F
Cell counts
Protein expression
Anti HA
Anti b2m
Anti HLA-A,B,C
BG control
A*02:01
A*11:01
B*35:01
C*12:03
HLA expression (Anti HLA-A,B,C - AF488)
Indicator binding (SA - AF647)
Bio-KLT
Bio-AIF
Bio-RYP
Bio-FAF
TCR-T/PC-CAR-T Target Antigens
Cancer Cell Line Encyclopedia
Cell Line
Cancer of origin
Fibrosarcoma
Rhabdomyosarcoma, alveolar
Rhabdomyosarcoma
Rhabdomyosarcoma, embryonal
Malignant Rhabdoid Tumor
Z-score
Differentially Up-regulated Surfaceome Genes in STS – Tang et al.
Mean Protein Abundance Log2 (FoT + 1)
Mean Log2 (CPM + 1) – scRNA
LMS
SS
MFS
UPS
DDLPS
WDLPS
WDLPS + DDLPS (mean)
DDLPS + WDLPS
Protein Expression
≥ 50th percentile
< 50th percentile

Fig S9. **Validation of peptide-MHC targets and protein to RNA correlation in the Tang et al. dataset.**

(A) Histograms showing the mean fluorescence intensity (MFI) for antibodies binding the HA tag, soluble β2m, and either HLA-A*02:01, A*11:01, B*35:01, or C*12:03, used in the IMMUNE workflow. **(B)** Scatter plots showing MFI for binding indicator to either HLA-A*02:01, A*11:01, B*35:01, or C*12:03, for the control peptides KLTPLCVTL, AIFQCSMTK, RYPVCKFPSL, and FAFCRITSF. Peptides with the highest binding for a given HLA allele were used as positive controls to assess the HLA binding of test peptides, as shown in Figure 5B. **(C)** Dot plot showing protein expression in STS cell lines in the CCLE database. **(D)** Scatter plots showing Spearman correlation between single cell RNA and protein expression in the Tang et al. STS proteomics dataset for LMS, UPS, SS, MFS, DDLPS, WDLPS. The genes plotted for each sarcoma histotype are the up-regulated surfaceome genes for each histotype that are also measured in the Tang et al. dataset. RNA expression is the average $\log_2$(CPM+1) across all tumor cells for a given STS histotype. Protein expression is reported as the average $\log_2$(FoT+1) protein abundance across all samples for a given STS histotype. **(E)** Scatter plots showing Spearman correlation between single cell RNA and protein expression in the Tang et al. STS proteomics dataset for both WDLPS and DDLPS, considering the differentially up-regulated surfaceome genes in liposarcoma (LPS). RNA expression is the average $\log_2$(CPM+1) across all LPS cells in our scRNA-seq data. Protein expression is reported as the average $\log_2$(FoT+1) protein abundance across all DDLPS and WDLPS samples. **(F)**. Scatter plot showing Spearman correlation analysis between single cell RNA expression and protein abundance in the Tang et al. STS proteomics dataset for both WDLPS and DDLPS, considering only the monospecific CAR targets defined in LPS. RNA expression is the average $\log_2$(CPM+1) across all LPS cells in the scRNA-seq dataset. Protein expression is reported as the average $\log_2$(FoT+1) protein abundance across all

DDLPS and WDLPS samples. Genes are separated based on the median protein abundance.

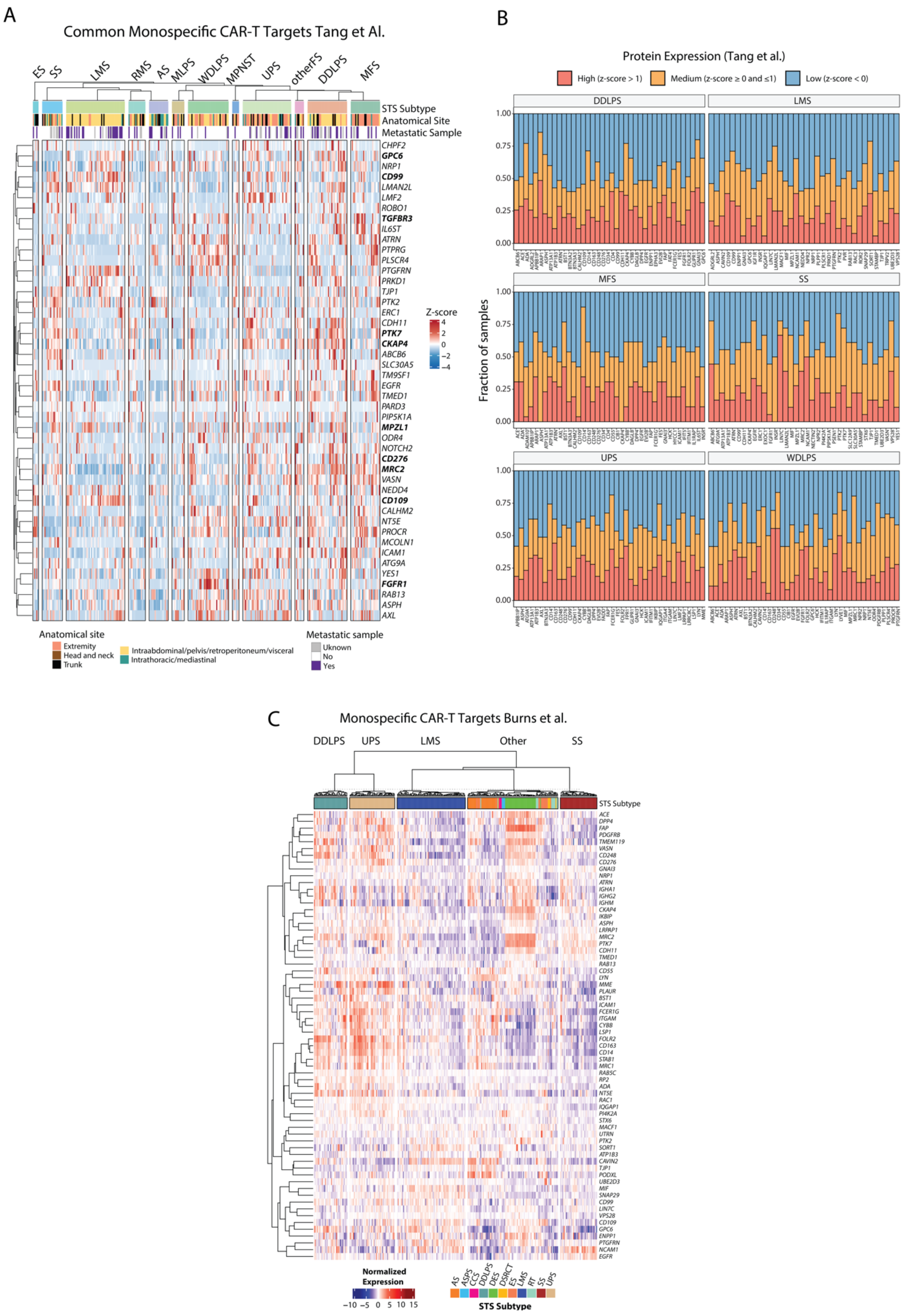
A
Common Monospecific CAR-T Targets Tang et Al.
ES
SS
LMS
RMS
AS
MLPS
WDLPS
MPNST
UPS
otherFS
DDLPS
MFS
STS Subtype
Anatomical Site
Metastatic Sample
CHPF2
GPC6
NRP1
CD99
LMAN2L
LMF2
ROBO1
TGFBR3
IL6ST
ATRN
PTPRG
PLSCR4
PTGFRN
PRKD1
TJP1
PTK2
ERC1
CDH11
PTK7
CKAP4
ABCB6
SLC30A5
TM9SF1
EGFR
TMED1
PARD3
PIP5K1A
MPZL1
ODR4
NOTCH2
CD276
MRC2
VASN
NEDD4
CD109
CALHM2
NT5E
PROCR
MCOLN1
ICAM1
ATG9A
YES1
FGFR1
RAB13
ASPH
AXL
Z-score
4
2
0
−2
−4
Anatomical site
Extremity
Head and neck
Trunk
Intraabdominal/pelvis/retroperitoneum/visceral
Intrathoracic/mediastinal
Metastatic sample
Uknown
No
Yes
B
Protein Expression (Tang et al.)
High (z-score > 1)
Medium (z-score ≥ 0 and ≤1)
Low (z-score < 0)
DDLPS
LMS
MFS
SS
UPS
WDLPS
Fraction of samples
0.00
0.25
0.50
0.75
1.00
C
Monospecific CAR-T Targets Burns et al.
DDLPS
UPS
LMS
Other
SS
STS Subtype
ACE
DPP4
FAP
PDGFRB
TMEM119
VASN
CD248
CD276
GNAI3
NRP1
ATRN
IGHA1
IGHG2
IGHM
CKAP4
IKBIP
ASPH
LRPAP1
MRC2
PTK7
CDH11
TMED1
RAB13
CD55
LYN
MME
PLAUR
BST1
ICAM1
FCER1G
ITGAM
CYBB
LSP1
FOLR2
CD163
CD14
STAB1
MRC1
RAB5C
RP2
ADA
NT5E
RAC1
IQGAP1
PI4K2A
STX6
MACF1
UTRN
PTK2
SORT1
ATP1B3
CAVIN2
TJP1
PODXL
UBE2D3
MIF
SNAP29
CD99
LIN7C
VPS28
CD109
GPC6
ENPP1
PTGFRN
NCAM1
EGFR
Normalized Expression
−10
−5
0
5
10
15
AS
ASPS
CCS
DDLPS
DES
DSRCT
ES
LMS
RT
SS
UPS
STS Subtype

Fig S10. Expression of monospecific CAR-T targets in the Tang et al. and Burns et al. STS clinical proteomics datasets.

(A) Protein expression in the Tang et al. dataset for monospecific CAR-T targets common to all histotypes. Expression is z-scored and median-centered on the whole dataset. Notable genes are in bold. **(B)** Stacked bar chart shows the fraction of samples with high (z-score > 1), medium ($0 \leq$ z-score ≤ 1), and low (z-score < 0) expression in the DDLPS, WDLPS, MFS, UPS, LMS, and SS samples of the Tang et al. STS dataset for the monospecific CAR targets. Only the top 40 proteins in each histotype with fraction of samples with medium and high expression $\geq$ 40% are shown. **(C)** Protein expression in the Burns et al. dataset for all the monospecific STS CAR-T targets identified. Expression is normalized through z-scoring and median-centering. MPNST: malignant peripheral nerve sheath tumor; MLPS: mucinous liposarcoma; others: other fibrosarcoma; ES: Ewing sarcoma; RMS: rhabdomyosarcoma; AS: angiosarcoma; ASPS: alveolar soft part sarcoma; CCS: clear cell sarcoma; DES: desmoid tumor; DSRCT desmoplastic small round cell tumor; RT: rhabdoid tumor.
